# Immunological effects of behavioral activation with exercise in major depression: an exploratory randomized controlled trial

**DOI:** 10.1038/tp.2017.76

**Published:** 2017-05-16

**Authors:** F Euteneuer, K Dannehl, A del Rey, H Engler, M Schedlowski, W Rief

**Affiliations:** 1 Division of Clinical Psychology and Psychotherapy, Department of Psychology, Philipps University of Marburg, Marburg, Germany; 2 Research Group Immunophysiology, Institute of Physiology and Pathophysiology, Philipps University of Marburg, Marburg, Germany; 3 Institute of Medical Psychology and Behavioral Immunobiology, University Clinic Hospital Essen, University of Duisburg-Essen, Essen, Germany

## Abstract

Major depression (MD) is associated with peripheral inflammation and increased cardiovascular risk. Regular physical exercise can have anti-inflammatory effects. The present study examined whether behavioral activation with exercise affects inflammatory processes in MD. Ninety-eight patients with MD were randomly assigned to cognitive-behavioral therapy (CBT) emphasizing exercise during behavioral activation (CBT-E), CBT with pleasurable low-energy activities as an active control condition (CBT-C) or a passive waiting list control group (WL). Plasma levels of C-reactive protein (CRP), interleukin (IL)-6, IL-10, lipopolysaccharide (LPS)-stimulated IL-6 production, and blood immune cell counts were analyzed at baseline and weeks 8 (post-behavioral activation) and 16 (post-treatment). Thirty non-depressed age- and sex-matched controls were included to examine potential immunological alterations in MD at baseline. Patients with MD exhibited higher levels of CRP, higher neutrophil and monocyte counts, lower IL-10 levels and reduced LPS-stimulated IL-6 production compared to controls (*P*<0.001−0.045). Multilevel modeling indicated that CBT-E was associated with increased anti-inflammatory IL-10 at weeks 8 and 16 compared to CBT-C and WL (*P*=0.004−0.018). CBT-E did not significantly affect other immunological makers in the total sample. A subgroup analysis including patients with potentially higher cardiovascular risk (CRP ⩾1 μg ml^−1^) indicated that CRP was reduced in CBT-E compared to CBT-C (*P*<0.007) and marginally reduced compared to WL (*P*<0.085) after week 16. The present findings provide new insights into immunological effects of behavioral treatments against depression. Behavioral activation in conjunction with exercise may have the potential to reverse, in part, immunological alterations in MD.

## Introduction

Major depression (MD) is associated with low-grade inflammation as evident from elevated levels of pro-inflammatory cytokines (for example, interleukin (IL)-6, tumor necrosis factor (TNF)-α) and acute-phase proteins (that is, C-reactive protein (CRP)) in the circulation.^1, 2^ Longitudinal observations in humans and studies in experimental animals document a bidirectional loop in which peripheral inflammatory signals can induce depressive symptoms and vice versa.^3, 4^ Mechanistically, elevated peripheral inflammatory signaling leads to dysregulation of several mood-relevant neural processes via molecular, cellular and neural routes.^5^ In this context, it has been suggested that reduced levels of anti-inflammatory cytokines, in particular IL-10, might also increase the risk for depression.^6^ Although the findings regarding the role of IL-10 in patients with MD are currently inconsistent,^1, 7, 8^ there is strong evidence from animal research suggesting an inverse relationship between IL-10 and depression-like behavior.^6, 9, 10, 11, 12^ In addition, a genetic predisposition toward producing low levels of IL-10 has been related to depressive symptoms.^13^ On the basis of the functional relation between depressive symptoms and cardiovascular disease (CVD), peripheral inflammatory pathways are considered a possible link between both clinical conditions.^14, 15, 16^

Physical inactivity is associated with both inflammation and depression.^17, 18, 19, 20^ Meta-analyses suggest that exercise (that is, regular, planned and structured physical activity) has moderate to large antidepressant effects^21, 22^ and even reduces depressive symptom severity in patients who did not remit with pharmacological antidepressant treatment.^23^ In addition, exercise induces anti-inflammatory effects mainly via the release of anti-inflammatory cytokines, such as IL-10, and has thus been suggested as a beneficial treatment option for clinical conditions related to inflammation.^24, 25, 26, 27, 28, 29, 30^ Although several studies document the anti-inflammatory effects of exercise in healthy individuals and patients with medical illness,^24^ little research focuses on the impact of physical activity on immunity in patients with clinically-relevant depressive symptoms. One trial found no significant effects of exercise treatment on circulating pro-inflammatory cytokine levels in a subgroup of patients with MD who were currently taking selective serotonin reuptake inhibitors (SSRIs), but were not responding to this treatment.^31^ When considering this finding, it should be noted that physical activity may overlap, in part, with some of the potential mechanisms of SSRIs.^22, 31, 32^ Thus, the potential of exercise to affect immunological markers may be attenuated in pharmacological non-responders.

Cognitive-behavioral therapy (CBT) is a standard treatment for depression^33^ and can be modified to increase exercise in the course of behavioral activation.^34, 35, 36, 37^ This randomized controlled trial examined whether CBT that emphasizes exercise during behavioral activation affects immunological markers in patients with MD. Immunological measures included plasma levels of CRP, IL-6 and IL-10, as well as *ex vivo* lipopolysaccharide (LPS)-stimulated IL-6 production and circulating leukocyte subpopulations. Although we expected that CBT in conjunction with exercise might be associated with a decrease in systemic inflammation (that is, CRP) and an increase in anti-inflammatory IL-10, this trial was considered exploratory. This is because we assessed potential treatment effects on a wide range of immunological markers, including those with inconsistent findings in MD such as leukocytes^38, 39^ as well as mitogen-stimulated cytokines, which has been shown to be increased,^40, 41, 42^ decreased^43, 44, 45, 46, 47, 48^ or unaltered^49, 50, 51^ in MD.

## Materials and Methods

### Participants

**Figure 1:**
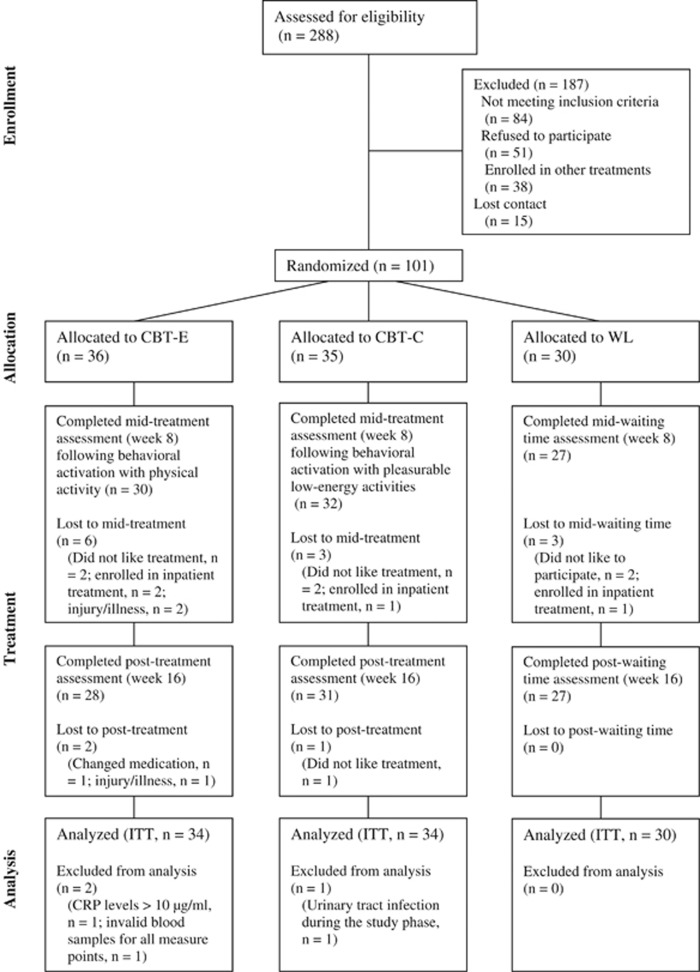
Flow of participants through each stage of the trial. CBT-C, cognitive-behavioral therapy control condition; CBT-E, cognitive-behavioral therapy with physical activity; CRP, C-reactive protein; ITT, intention-to-treat; WL, waitlist control group.

This randomized controlled trial was conducted from August 2011 to February 2015 with German Psychological Society Review Board approval. The study was part of the Outcome of Psychological Interventions in Depression (OPID) trial. OPID is an ongoing research project that aims to improve outcomes in treatment for MD. OPID involves four different arms: (i) CBT with exercise (CBT-E), (ii) an active control condition for CBT-E, including CBT with pleasurable low-energy activities (CBT-C), (iii) Cognitive Behavioral Analysis System of Psychotherapy and (iv) a passive waitlist control condition (WL). Arms (i–iii) also captured a comprehensive immunological evaluation and were funded as a separate subproject by the German Research Foundation from 2011–2015 (DFG RI 574/23-1/SCHE 341/20-1; ‘Effects of psychotherapy with physical activity on inflammatory markers in patients with major depression’). Power calculations^52^ to provide estimates for the necessary sample size for F-tests were conducted concerning CRP as a primary outcome for systemic inflammation. To detect a medium effect of group × time, with a statistical power of 1−*β*=0.80 and a level of significance of *α*<0.05, a sample size of at least 86 needs to be included when assuming that correlations between repeated measures are moderate.^53^ Considering lower stability as well as partially unknown and potentially smaller effect sizes for cytokines and immune cell counts, a total sample size of *N*=150 was targeted, but not reached due to the conclusion of funding and a slower than anticipated recruitment into the trial. Ninety-eight patients aged 18–65 who fulfilled criteria for MD in DSM-IV^54^ and who were randomly assigned using simple computerized randomization to either CBT-E, CBT-C or WL were analyzed (see Figure 1 for study flow). A sample of 30 age- and sex-matched healthy controls from the same community was studied to examine potential baseline alterations in immunological markers in MD. Patients were recruited via the Outpatient Clinic for Psychological Interventions of the University of Marburg, via advertisements, leaflets in pharmacies and waiting rooms of doctors, as well as press releases in local newspapers. Healthy controls were recruited via advertisements and press releases in local newspapers. After prescreening via phone, participants underwent a diagnostic session that included the German version of the structured clinical interview for DSM-IV^55^ and an interview that focused on exclusion criteria and socio-demographic variables. Exclusion criteria were neurological illness, psychotic symptoms, injuries and infections during the last 14 days, alcohol and/or drug abuse, antipsychotics, stimulants, current pregnancy and lactation in women, and any mental disorders according to DSM-IV for healthy controls. Patients who took antidepressants were considered for participation under the assumption that the dose had been stable for at least 2 weeks and would remain so during study participation. Informed written informed consent was obtained from all participants.

### Interventions

Both CBT treatments (that is, CBT-E and CBT-C) were based on a common CBT manual and structured through phases typically used in CBT.^56^ Patients participated in 50 min of individual manualized psychotherapy weekly for 16 weeks. All therapists were clinical psychologists with advanced or completed postgraduate clinical training in CBT. Patients and therapists were blinded to the purpose and study hypothesis. After an initial phase (weeks 1–4), patients received behavioral activation (weeks 5–9) with either exercise (CBT-E) or pleasant low-energy activities in the active control condition (CBT-C), followed by cognitive therapy (weeks 10–16).

For CBT-E, CBT was modified to increase physical activity according to the recommendations of the World Health Organization.^57^ During the initial phase, patients received psychoeducation on MD and on the relationship between thoughts, feelings and behavior, with a focus on physical activity as a health behavior potentially relevant for depressive symptoms.^20, 58^ Psychoeducation further addressed recommendations for being physically active.^57, 58^ Additional elements were case conceptualization (that is, assessment of individual risk factors for depression) and, if necessary, problem-solving strategies were applied to reduce barriers to physical activity (for example, coping with low social support, arranging options for exercise). Patients received a manual summarizing the content of psychoeducation and providing a list of potential physical activities (for example, walking, jogging, swimming, gyms), as well as physical activity dose recommendations based on the Ainsworth Compendium of Physical Activities.^59^ These issues were discussed within treatment sessions and used to prepare an individualized schedule with at least four 40-min homework exercise sessions per week, consisting of at least moderate physical activity. During the phase of behavioral activation, the schedule was applied and common behavioral activation techniques were used to assist patients (for example, reinforcement, activity and mood monitoring, problem solving). After the phase of behavioral activation, therapists prescribed patients to continue physical activity, but shifted their focus to cognitive aspects, such as modification of dysfunctional cognitions and beliefs, enhancement of cognitions that increase psychological well-being, as well as prevention of relapses.^56^

The active control condition (that is, CBT-C) involved CBT with behavioral activation emphasizing pleasurable experiences without a substantial increase in physical activity (that is, euthymic activities). Different from CBT-E, patients received psychoeducation on MD and on the relationship between thoughts, feelings and behavior with a focus on euthymic activities. The euthymic activities were based on a manual for euthymic therapy, an intervention for mental disorders that shares similarities with mindfulness therapy.^60^ Analogous to CBT-E, the activity schedule within behavioral activation involved at least four 40-min homework sessions per week including euthymic exercises that bring awareness to different senses such as hearing (for example, listening to music), tasting (for example, preparing and enjoying a meal), smelling (for example, taking a scented bath) or touching (for example, bringing attention to the sensations of skin contact with pleasant surfaces).^60^ After the phase of behavioral activation, therapists also prescribed patients to perform euthymic activities autonomously and shifted the focus to a cognitive therapy similar to CBT-E. Patients in the WL condition (that is, passive control condition) did not receive any treatment, but were involved in regular psychotherapy after their 16-week waiting time.

### Depressive symptoms and physical activity measures

Self-reported outcomes included depressive symptoms as assessed by the German version of the Beck Depression Inventory-II^61^ and metabolic equivalent minutes per week (MET-min per week) for three domains of physical activity (walking, moderate-intensity and vigorous-intensity), as assessed by the long version of the International Physical Activity Questionnaire (IPAQ).^62^

### Immunological measures

Before each blood sampling, participants were queried about acute infections during the last 14 days, chronic infections or illness, and a sample was considered missing if participants reported any one of these issues. Participants were instructed to avoid exercise and alcohol 24 h prior to blood withdrawal. Non-fasting blood samples were collected in EDTA-treated or heparinized tubes (S-Monovette, Sarstedt, Nümbrecht, Germany) between 0700 hours and 1000 hours. Plasma for CRP and cytokine measurements were separated by centrifugation at 2,000 *g* for 10 min at 4 °C, and plasma was stored at –80 °C (7 to 12 months) until analysis. CRP was measured using an enzyme-linked immunosorbent assay (CRP high-sensitive ELISA, IBL International, Hamburg, Germany) according to the manufacturer’s instructions. Plasma levels of IL-6 and IL-10 were analyzed by flow cytometry using bead-based assays (Bio-Plex Pro Human Cytokine Assays, Bio-Rad Laboratories, Hercules, CA, USA) as previously described.^63^ The sensitivity of the assays was 0.02 μg ml^−1^ for CRP, 0.45 pg ml^−1^ for IL-6 and 0.59 pg ml^−1^ for IL-10. Complete blood counts including the white blood cell differential were obtained using an automated hematology analyzer (XT-2000i, Sysmex, Horgen, Switzerland). Leukocyte subpopulations were determined by flow cytometry using a standard lyse/wash procedure and the following antibodies (all from BioLegend, San Diego, CA, USA): FITC-conjugated anti-human CD3 (clone SK7), Pacific Blue-conjugated anti-human CD4 (clone SK3), PE-Cy7-conjugated anti-human CD8 (clone SK1), APC-Cy7-conjugated anti-human CD14 (clone M5E2), PerCP-Cy5.5-conjugated anti-human CD19 (clone HIB19), PE-conjugated anti-human CD25 (clone BC96), PE-conjugated anti-human CD56 (clone MEM-188) and AF647-conjugated anti-human CD127 (clone A019D5). Samples were analyzed on a FACSCanto II flow cytometer (BD Biosciences, Heidelberg, Germany) using BD FACSDiva software (Version 8.0.1, BD Biosciences). For the assessment of *ex vivo* IL-6 production, heparinized blood was diluted 1:5 with cell culture medium (RPMI 1640, Invitrogen, Karlsruhe, Germany, containing 10% fetal calf serum, PAA, Cölbe, Germany, and Gentamicin 50 μg ml^−1^, Invitrogen, Karlsruhe, Germany) and stimulated with 5 μg ml^−1^ LPS from *E. coli* 0111:B4 (Sigma-Aldrich, Taufkirchen, Germany) in 24-well flat-bottom microtiter plates. After incubation (72 h, 37 °C, 5% CO_2_),^64^ culture supernatants were collected by centrifugation (300 *g*, 4 °C, 5 min) and stored at −80 °C (7 to 12 months) until analysis. IL-6 concentration in the supernatants was measured using a commercial ELISA (ELISA MAX Deluxe, BioLegend) according to the manufacturer’s instructions. The sensitivity of the assay was 4 pg ml^−1^.

### Data analysis

Statistical analyses were carried out with SPSS version 20.0 for Windows (SPSS, Chicago, IL, USA). Baseline differences in group characteristics were calculated using pairwise comparisons with *t*-tests, if necessary with Welch’s correction, and *χ*^2^ tests. Intervention effects on outcomes were analyzed on an intention-to-treat basis using multilevel models (MLM).^65^ MLMs were tested with different covariance structures, and for each MLM, the covariance structure that provided the best fit was selected.^66, 67^ Missing values occurred, and extreme outliers in immunological variables were also considered missing values (that is, values more than three interquartile ranges above the 75th percentile).^68^ Among those who completed the interventions, the overall data were available as follows: BDI-II (87%), IPAQ (88%), CRP (89%), IL-6 (86%), IL-10 (77%), LPS-stimulated IL-6 (69%) and leukocyte counts and subsets (83%). Data from 98 randomized participants were analyzed in MLMs (see Figure 1 for study flow). Full information maximum likelihood estimation was used to handle missing data. Simulations suggest that multiple imputation does not contribute to the precision of MLMs and that full information maximum likelihood might be more appropriate to receive unbiased results.^69, 70^ Given the exploratory nature of this study, analyses were not corrected for multiple testing.^71^ However, in cases of significant (*P*<0.05) treatment effects only (that is, group × time interactions), *post hoc* contrasts were calculated to specify these effects by testing group differences (that is, CBT-E versus CBT-C versus WL) at week 8 (mid-treatment, post-behavioral activation) and at week 16 (post-treatment). Log transformation was applied if skewed data could theoretically be a concern.^68, 72^

An *a priori* subgroup analysis was defined for CRP. Unlike other inflammatory markers, longitudinal research on CRP has resulted in a position statement recommending cutoff levels of CRP <1, 1–3 and >3 μg ml^−1^ equating to low, intermediate and high risk for subsequent CVD.^73^ Accordingly, it has been suggested to reduce circulating CRP in case of values ⩾1 μg ml^−1^.^74^ Given the potential clinical relevance of this classification, we ran an *a priori* subgroup analysis for participants with elevated CRP (CRP⩾1 μg ml^−1^) to focus on patients who may have had at least intermediate CRP-associated cardiovascular risk and might thus have benefited from CRP reduction.^73^

## Results

### Baseline characteristics and psychometric outcomes

A total of 101 patients with MD underwent baseline assessment, and 86 (85%) completed the assigned interventions (see Figure 1 for study flow). Three participants were excluded because their CRP levels were above 10 μg ml^−1^ at all measure points (that is, indicating acute inflammation; *n*=1), invalid blood samples for almost all measures (*n*=1), or a retrospective report showed chronic urinary tract infection during the study phase (*n*=1). Descriptive statistics for all groups and comparisons between patients with MD and age- and sex-matched healthy controls are presented in Table 1. Compared to controls, patients with MD showed higher levels of CRP (*t*_110.5_=4.01, *P*<0.001), lower levels of circulating IL-10 (*t*_30.8_=−2.09; *P*=0.045), as well as a higher IL-6/IL-10 ratios (*t*_84.1_=2.71, *P*=0.008). Neutrophil counts (*t*_39.5_=2.15; *P*=0.038) and monocyte counts (*t*_39.5_= 2.15; *P*=0.006) were higher in MD than in healthy controls. The LPS-stimulated IL-6 production was lower in MD than in healthy controls (*t*_86.3_=−2.89; *P*=0.005). There were no significant differences between groups for any other immunological measures (*P*=0.061−0.924).

Table 2 illustrates outcomes from baseline to week 8 (that is, mid-treatment, post-behavioral activation) and week 16 (that is, post-treatment) by treatment group. The profile of change over time between the three groups was statistically significant for depressive symptoms (group × time: F_4,81.3_=6.27; *P*=<0.001). As compared with WL, CBT-E was associated with significantly lower depressive symptoms at week 8 (*t*_95.1_=2.88; *P*=0.005) and week 16 (*t*_86.9_=2.90; *P*=0.005). As compared with WL, CBT-C was also related with lower depressive symptoms at week 8 (*t*_94_= 3.15; *P*=0.002) and 16 (*t*_83_=3.16; *P*=0.003). Depressive symptoms were not significantly different between CBT-E and CBT-C at week 8 (*t*_74.7_=0.23; *P*=0.816) and 16 (*t*_86.5_=−0.14; *P*=0.889).

Change in vigorous-intensity activity in the three groups from baseline to week 8 and week 16 was statistically significant (group × time: F_4,78.7_=3.25; *P*=0.016). As compared to WL, CBT-E was associated with higher levels of vigorous-intensity activity at week 8 (*t*_90.4_=−2.00; *P*=0.049) with non-significant differences at week 16 (*t*_78.3_=−1.51; *P*=0.136). As compared to CBT-C, CBT-E was related with higher levels of vigorous-intensity activity at week 8 (*t*_72.9_=−2.74; *P*=0.008) and a trend for higher levels at week 16 (*t*_78.4_=−1.80; *P*=0.075). WL and CBT-C resulted in no statistically significant difference in vigorous-intensity activity at week 8 (*t*_90_=0.12; *P*=0.907) and 16 (*t*_76.7_=0.25; *P*=0.801). There were no statistically significant group × time interactions on either walking or moderate-intensity activity (*P*=0.358−0.499).

### Immunological outcomes

**Figure 2:**
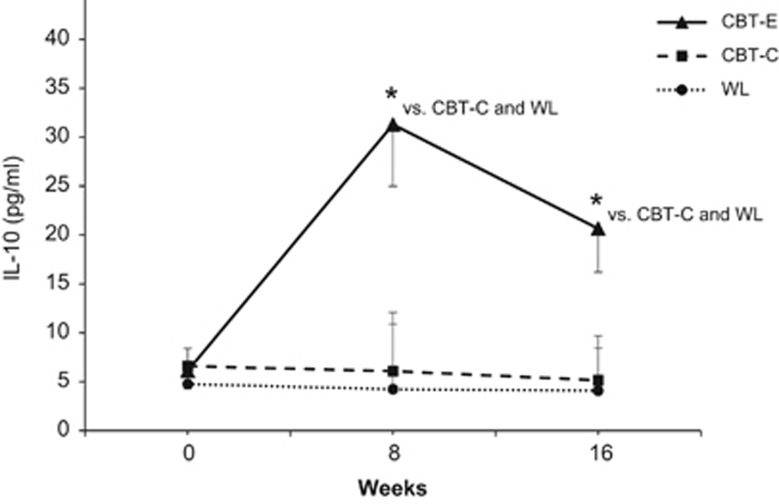
Circulating levels of anti-inflammatory interleukin-10 (IL-10) from baseline to week 8 (post-behavioral activation), and to week 16 (post-treatment) by treatment group. Values are estimated marginal means (s.e.m.) from multilevel modeling. Pairwise contrasts for cognitive-behavioral therapy with exercise (CBT-E) versus cognitive-behavioral therapy with pleasurable low-energy activities (CBT-C, active control condition) versus waitlist (WL, passive control condition): *P<0.05.

Changes in levels of CRP, pro-inflammatory cytokines and immune cell counts from baseline to week 8 and week 16 were not significantly different between groups (*P*=0.068−0.711) (Table 2). However, the profile of change over time between the three groups was statistically significant for anti-inflammatory IL-10 (group × time: F_4,157.7_=3.88; *P*=0.005) (Figure 2). Contrasts indicated that patients in the CBT-E group had higher levels of IL-10 at week 8 (*t*_69_=−2.96; *P*=0.004) and 16 (*t*_66_=−2.66; *P*=0.010) than patients in the WL condition. Compared to CBT-C, CBT-E was also related to higher levels of IL-10 at week 8 (*t*_69_=−2.88; *P*=0.005) and 16 (*t*_66_=−2.43; *P*=0.018). CBT-C and WL resulted in no statistically significant differences in IL-10 at week 8 (*t*_69_=−0.20; *P*=0.837) and 16 (*t*_66_=−0.17; *P*=0.866).

**Figure 3:**
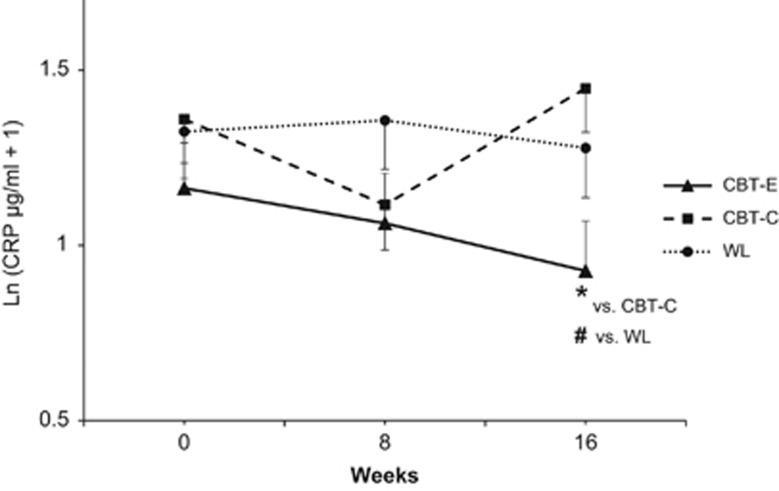
Subgroup analysis (*N*=42) of patients with potentially elevated cardiovascular risk (CRP ⩾1 μg ml^−1^). Circulating levels of C-reactive protein (CRP) from baseline to week 8 (post-behavioral activation), and to week 16 (post-treatment) by treatment group. Values are estimated marginal means (s.e.m.) from multilevel modeling. Pairwise contrasts for cognitive-behavioral therapy with exercise (CBT-E) versus cognitive-behavioral therapy with pleasurable low-energy activities (CBT-C, active control condition) versus waitlist (WL, passive control condition): *P<0.05, ^#^*P*<0.1.

Forty-two patients with MD had elevated levels of CRP (CRP ⩾1 μg ml^−1^) at baseline and were thus included in the predefined subgroup analysis of participants with a potentially increased risk for CVD. Given the smaller sample size with respect to the total subgroup and groups for contrast calculations, the central limit theorem was not applied and log-transformation with a constant of 1 was used to reduce skewness.^68^ For this subgroup, the profile of change indicated that CBT-E reduces levels of CRP (group × time: F_4,71.6_=2.60; *P*=0.043) (Figure 3). As compared with WL, CBT-E resulted in a trend of lower CRP levels at week 16 (*t*_83.2_= 1.75; *P*=0.085), but not at week 8 (*t*_88.3_=1.47; *P*=0.145). As compared with CBT-C, CBT-E was associated with lower CRP levels at week 16 (*t*_80_= 2.75; *P*=0.007), but not at week 8 (*t*_88.3_=0.28; *P*=0.783). WL and CBT-C resulted in no statistically significant differences in CRP levels at week 8 (*t*_79.3_=−0.89; *P*=0.374) and 16 (*t*_83.5_=1.27; *P*=0.209).

## Discussion

Depression is associated with inflammation and increased cardiovascular risk.^1, 2, 15^ As there is evidence that physical activity can induce anti-inflammatory effects,^27, 29, 75, 76^ this randomized controlled trial examined the impact of CBT, emphasizing behavioral activation with exercise (that is, CBT-E), on immunological markers in patients with MD. At study entry, patients with MD showed decreased peripheral levels of anti-inflammatory IL-10 and increased levels of CRP compared to healthy controls, corroborating previous findings.^2, 7, 8^ CBT-E induced a significant increase in IL-10 plasma concentrations. This observation is novel in the context of depression and complements findings from other clinical conditions.^27, 28, 30, 77^ In addition, CBT-E reduced levels of CRP among those patients with potentially elevated cardiovascular risk (CRP⩾1 μg ml^−1^), confirming data from previous exercise trials.^26, 27, 29, 76^

For the total sample of patients in this study, there was no evidence for an effect of CBT with exercise on immunological markers with the exception of IL-10. One possible pathway of exercise-induced regulation of immunological mediators is a temporary increase of catecholamines resulting in activation of beta-adrenergic receptors on monocytes.^78, 79, 80^ Depressive symptoms are associated with reduced sensitivity of beta-adrenergic receptors.^81^ Thus, it is possible that the potential of exercise to affect immunological processes is reduced in patients with depression.^39^ However, IL-10 release is also mediated by beta-adrenergic pathways,^82^ and we found evidence for an effect of physical activity on CRP in a subsample with elevated CRP baseline levels. Thus, our findings may simply demonstrate that an anti-inflammatory effect of exercise is relevant for a subpopulation of patients with depression. As pointed out by Kiecolt-Glaser *et al.*,^5^ inflammation may neither be necessary nor sufficient to induce or sustain depression in general, but it is relevant for a substantial subpopulation of depressed individuals. Thus, observable reductions in inflammatory markers due to anti-inflammatory interventions may only be expected in this subpopulation, as observed in our subsample with increased baseline levels of CRP.

A limited number of studies so far analyzed the impact of CBT on immune functions. For example, a recent randomized controlled trial reported that CBT reduces levels of CRP in insomnia,^83^ a condition which is frequently associated with depression.^84, 85^ In contrast, two pre-post studies found no reduction in CRP levels during CBT in patients with depression, although one of these studies reported decreased expression of Toll-like receptors after CBT.^86, 87^ CBT mainly consists of two core components: behavioral activation and cognitive therapy.^88^ When applying behavioral activation, individuals may increase both physical activity and pleasurable experiences. Thus, it is important to note that this study also dismantles behavioral activation within CBT, in (1) behavioral activation with physical activity (that is, CBT-E), and (2) behavioral activation with pleasurable experiences without a substantial increase in physical activity (that is, CBT-C). In view of the observed pattern of changes in this study, a potential anti-inflammatory effect of CBT might be strengthened when explicitly focusing on physical activity^75^ during behavioral activation.

Patients with depression are at increased risk for CVD.^14, 15, 16^ Although the prospective value of CRP for CVD is well established,^73^ the clinical relevance of IL-10 is less clear,^89, 90, 91^ in particular for patients with depression. *In vitro* and *in vivo* studies in animals clearly demonstrate an atheroprotective role of IL-10.^92^ To the best of our knowledge, no longitudinal studies have examined the link between IL-10 and future health outcomes in patients with depression without ‘medical illness’. However, Parissis *et al.*^93^ studied the prognostic value of IL-10 and other biomarkers in patients with chronic heart failure (CHF) and comorbid clinically-relevant depressive symptoms. Similar to our findings, CHF patients with depressive symptoms had lower levels of circulating IL-10 than non-depressed CHF patients. Other than several pro-inflammatory markers, lower levels of IL-10 independently predicted major adverse cardiovascular events during a period of 1 year. Thus, longitudinal studies investigating the prognostic value of IL-10 in depressed patients without manifest baseline CVD are warranted to evaluate the clinical relevance of our findings.

MD patients showed significantly increased neutrophil and monocyte numbers, as well as significantly reduced IL-6 production after *ex vivo* LPS-stimulation at baseline. The clinical relevance in the context of MD is also unclear as data on numbers of circulating neutrophils and monocytes in MD,^38, 94, 95^ and findings from studies of mitogen-stimulated cytokine production are inconsistent and controversial.^41, 42, 44, 45, 46, 47, 48^ As LPS or mitogens-stimulated production of cytokines is not representative of systemic inflammation,^2, 47, 96^ a reduced *ex vivo* production of cytokines in MD patients may reflect an exhaustion of cell function subsequent to sustained systemic low-grade inflammation.^46, 47^

This study has several limitations. Given the exploratory nature of this trial, further research is necessary to confirm the observed findings. In addition, despite a sample size of 98 patients with MD, the number of subjects with elevated CRP was relatively small and thus our results need replication. Moreover, the total sample size was insufficient for detection of small effects. Our sample consisted of outpatients with MD who were eligible for psychological treatment. Thus, findings may not generalize to other samples of patients (for example, MD patients with psychotic features). Although the physical activity questionnaire used in this study has good reliability and validity,^97, 98^ response bias cannot be excluded and an additional use of objective measures would have been beneficial. Finally, despite the extensive data linking inflammation, in particular CRP, to cardiovascular risk, the clinical implications of the present and previous intervention studies are not clear. The most important question might be whether lowering CRP or increasing IL-10 in MD through interventions will translate into reduced risk for CVD. Given the lack of studies that focus on such long-term effects, it is not possible to answer this question. However, though clinical implications are speculative, our results indicate that an established risk marker for CVD (that is, CRP) and an anti-inflammatory cytokine with atheroprotective properties (that is, IL-10) can be influenced by behavioral treatments in MD.

This study demonstrates that CBT with exercise may have anti-inflammatory effects in patients with MD by increasing IL-10 and reducing CRP among those patients with increased levels of CRP and potentially elevated risk for CVD. Important strengths of this randomized controlled trial are the involvement of both an active and a passive control condition, a baseline comparison between patients with MD and healthy controls, as well as a broad assessment of immunological markers. The findings provide new insights into the immunological effects of behavioral treatments against depression. Given the links between depression, inflammation and CVD, this study introduces important implications regarding how behavioral treatments can be conceptualized to target inflammation and possibly promote cardiovascular health in MD.

## Figures and Tables

**Table 1:** Baseline characteristics of participants

*Variable*	*MD, CBT-E* (N=*34*)	*MD, CBT-C* (N=*34*)	*MD, WL* (N=*30*)	*MD, total* (N=*98*)	*HC* (N=*30*)	t *or* χ^2^ P*-value*
Age	36.9 (10.8)	37.2 (12.5)	37.9 (13.5)	37.3 (12.2)	37.1 (12.2)	0.08; 0.939
Female, number (%)	16 (47.1)	19 (55.9)	13 (43.3)	48 (49)	15 (50)	0.01; 0.922
Depressive symptom severity, BDI-II	27 (9.1)	27.3 (8.7)	26.2 (9.9)	26.9 (9.1)	4.6 (5.5)	18.26; <0.001

*DSM-IV Axis I comorbidity, number* (*%)*						
Anxiety disorders	8 (23.5)	7 (20.6)	8 (26.7)	23 (23.5)	0 (0)	8.58; 0.003
Somatoform disorders	2 (5.9)	4 (11.8)	6 (20)	12 (12.2)	0 (0)	4.05; 0.044
						
Body mass index, kg/m^2^	25.8 (4.1)	26.2 (6.3)	26.5 (5.7)	26.1 (5.3)	24 (4.2)	1.96; 0.052
Education (years)	11.5 (1.7)	11.3 (1.7)	11 (1.7)	11.3 (1.7)	12.2 (1.5)	−2.83; 0.006
Number of cigarettes per day	3.5 (7.3)	1.5 (4.8)	4.2 (8.8)	3 (7.1)	0.5 (1.9)	1.90; 0.060
Antidepressant medication, number (%)	14 (41.2)	13 (38.2)	10 (33.3)	37 (37.8)	0 (0)	15.93; <0.001

*Physical activity, IPAQ, MET-minutes per week*						
Walking	1405 (1646.6)	1269.9 (1255.9)	1425.1 (1266.5)	1364.8 (1392.5)	1919.5 (2020.6)	−1.61; 0.092
Moderate-intensity activity	1817.7 (2321)	2441.9 (2321.3)	1842.5 (1884.8)	2040 (2227)	2243.8 (2813.1)	−0.87; 0.388
Vigorous-intensity activity	917.5 (1561.1)	898.2 (1574.4)	760 (1921.1)	861.1 (1670.6)	1577.3 (1739.28)	−2.03; 0.045
						
CRP, μg ml^−1^	1.4 (1.6)	1.8 (1.9)	1.8 (2)	1.7 (1.8)	0.8 (0.6)	4.01; <0.001
CRP ⩾1 μg ml^−1^, number (%)	14 (41.2)	15 (44.1)	13 (43.3)	42 (42.9)	7 (23.3)	4.39; 0.036
IL-6, pg ml^−1^	4.6 (6.9)	3.7 (4.3)	3.6 (3.7)	3.9 (5.1)	5.9 (7.8)	−1.22; 0.230
IL-10, pg ml^−1^	6.1 (9.6)	6.6 (11.1)	4.8 (6.2)	5.8 (9.2)	12.9 (16.3)	−2.09; 0.045
IL-6/IL-10 ratio	0.9 (0.8)	0.9 (0.9)	1.2 (1)	1 (0.9)	0.6 (0.4)	2.71; 0.008
LPS-stimulated IL-6 production, pg ml^−1^	161.3 (153.2)	163 (116.2)	217.9 (143)	183.8 (138.5)	249.8 (84.1)	−2.89; 0.005

*Immune cell counts per μl*						
Leukocytes	7070 (2047)	6013 (1525)	7303 (1881)	6780 (1889)	6740 (2209)	0.69; 0.924
Lymphocytes	2193 (764)	2070 (531)	2199 (717)	2152 (669)	2286 (679)	−0.82; 0.413
Neutrophils	4319 (1376)	3479 (1174)	4535 (1510)	4099 (1420)	3520 (989)	2.15; 0.038
Monocytes	417 (143)	373 (134)	421 (122)	403 (134)	310 (153)	2.78; 0.006
Total T cells	1510 (616)	1368 (396)	1492 (601)	1454 (541)	1494 (460)	−0.31; 0.761
T helper cells	962 (450)	930 (435)	954 (412)	948 (427)	920 (372)	0.27; 0.789
Cytotoxic T cells	464 (247)	402 (122)	416 (182)	426 (188)	485 (147)	−1.34; 0.184
Regulatory T cells	83 (38)	92 (49)	86 (36)	87 (41)	73 (28)	1.93; 0.061
B cells	213 (79)	244 (131)	248 (137)	236 (147)	197 (94)	1.38; 0.169
NK cells	256 (152)	261 (152)	257 (143)	258 (147)	281 (128)	−0.65; 0.516

Abbreviations: BDI, Beck Depression Inventory; CBT-C, Cognitive-behavioral therapy control condition; CBT-E, Cognitive-behavioral therapy with exercise; CRP, C-reactive protein; DSM, Diagnostic and Statistical Manual of Mental Disorders; HC, healthy control group; IL, interleukin; IPAQ, International Physical Activity Questionnaire; LPS, lipopolysaccharide; MD, major depression; MET, metabolic equivalent; WL, waitlist control group.

Values are mean (s.d.) unless noted with percentage. Group differences were calculated using *χ*^2^ tests for categorical variables and analyses of variance or *t*-tests for continues variables.

**Table 2:** Outcomes from baseline to week 8 and week 16 by treatment group: descriptive statistics and group × time effect

	*Baseline*	*Week 8* *post-behavioral activation*	*Week 16* *post-treatment*	*MLM:* *group × time: F;* P*-value*
*Depressive symptom severity, BDI-II*				
CBT-E	27 (9.1)	18.4 (10.7)	14.6 (13.5)	
CBT-C	27.3 (8.7)	19.1 (9.5)	14.8 (11.4)	6.27, <0.001
WL	26.2 (9.9)	29.5 (12.1)	23.5 (11)	

*Walking, MET-minutes per week*				
CBT-E	1405 (1646.6)	996 (897.5)	1206.1 (1077)	
CBT-C	1269.9 (1255.9)	1030.5 (1002.4)	1131.9 (1112.5)	1.11, 0.358
WL	1425.1 (1266.5)	1245.8 (1168.2)	1923.5 (1566.9)	

*Moderate-intensity activity, MET-minutes per week*				
CBT-E	1817.7 (2321)	1981.5 (2667.4)	2396.5 (3812.8)	
CBT-C	2441.9 (2321.3)	1437.3 (1997.6)	1996.5 (2624.8)	0.84, 0.499
WL	1842.5 (1884.8)	1620 (1538.2)	1712.3 (1751.8)	

*Vigorous-intensity activity, MET-minutes per week*				
CBT-E	917.5 (1561.1)	1640 (2137.9)	1669.6 (2815.2)	
CBT-C	898.2 (1574.4)	549.7 (1153.3)	714.6 (1287.9)	3.25, 0.016
WL	760 (1921.1)	565.7 (786)	692.3 (1483.1)	

*CRP, μg ml^−1^*				
CBT-E	1.4 (1.6)	1.3 (1.1)	1.4 (1.1)	
CBT-C	1.8 (1.9)	1.3 (1.7)	2.4 (2.7)	1.78, 0.137
WL	1.8 (2)	2 (2.5)	2 (2)	

*IL-6, pg ml^−1^*				
CBT-E	4.6 (6.9)	9.5 (15)	8.1(11.5)	
CBT-C	3.7 (4.3)	4.5 (5.5)	4.7 (5.2)	2.12, 0.093
WL	3.6 (3.7)	2.8 (2.7)	2.8 (2.7)	

*IL-10, pg ml^−1^*				
CBT-E	6.1 (9.6)	31.3 (52.4)	20.6 (36.3)	
CBT-C	6.6 (11.1)	6.1 (9.6)	5.2 (5.5)	3.88, 0.005
WL	4.8 (6.2)	4.2 (5.1)	4.1 (5)	

*IL-6/IL-10 ratio*				
CBT-E	0.9 (0.8)	0.7 (0.6)	0.7 (0.6)	
CBT-C	0.9 (0.9)	1 (0.8)	1.3 (1.4)	0.85, 0.500
WL	1.2 (1)	1 (0.7)	1.1 (0.8)	

*LPS-stimulated IL-6 production, pg ml^−1^*				
CBT-E	161.3 (153.2)	194.5 (140.7)	175.2 (156.4)	
CBT-C	163 (116.2)	159.2 (118.1)	141.1 (885.1)	1.78, 0.138
WL	217.9 (143)	180.4 (115.5)	175.2 (156.4)	

*Leukocytes, cell counts per μl*				
CBT-E	7070 (2047)	7195 (2159)	7635 (1868)	
CBT-C	6013 (1525)	6289 (1959)	6388 (1495)	2.26, 0.068
WL	7303 (1881)	6478 (1562)	6630 (1612)	

*Lymphocytes, cell counts per μl*				
CBT-E	2393 (764)	2306 (797)	2441 (756)	
CBT-C	2070 (531)	2051 (592)	2168 (353)	2.09, 0.086
WL	2199 (717)	2065 (629)	2101 (562)	

*Neutrophils, cell counts per μl*				
CBT-E	4319 (1376)	4408 (1514)	4584 (1492)	
CBT-C	3479 (1174)	3605 (1127)	3816 (1212)	1.91, 0.114
WL	4535 (1510)	3926 (1142)	3893 (1299)	

*Monocytes, cell counts per μl*				
CBT-E	417 (143)	379 (156)	410 (133)	
CBT-C	373 (134)	354 (126)	358 (140)	0.53, 0.711
WL	421 (122)	369 (129)	350 (132)	

*Total T cells, cell counts per μl*				
CBT-E	1510 (616)	1616 (674)	1705 (607)	
CBT-C	1368 (396)	1349 (433)	1433 (272)	1.85, 0.123
WL	1492 (601)	1416 (521)	1466 (524)	

*T helper cells, cell counts per μl*				
CBT-E	962 (450)	952 (366)	1155 (449)	
CBT-C	930 (435)	842 (273)	931 (223)	1.85, 0.123
WL	954 (412)	944 (382)	978 (398)	

*Cytotoxic T cells, cell counts per μl*				
CBT-E	464 (247)	449 (223)	497 (249)	
CBT-C	402 (122)	407 (140)	430 (147)	1.29, 0.278
WL	416 (182)	422 (228)	395 (129)	

*Regulatory T cells, cell counts per μl*				
CBT-E	83 (38)	88 (33)	105 (39)	1.07, 0.372
CBT-C	92 (49)	72 (31)	85 (30)	
WL	86 (36)	82 (31)	88 (32)	

*B cells, cell counts per μl*				
CBT-E	213 (79)	240 (107)	230 (102)	
CBT-C	244 (131)	248 (97)	260 (92)	2.04, 0.099
WL	248 (137	230 (137	201 (114)	

*NK cells, cell counts per μl*				
CBT-E	256 (152)	204 (115)	306 (180)	
CBT-C	261 (152)	255 (137)	272 (122)	0.64, 0.637
WL	257 (143)	212 (100)	262 (114)	

Abbreviations: BDI, Beck Depression Inventory; CBT-C, cognitive-behavioral therapy control condition; CBT-E, cognitive-behavioral therapy with exercise; CRP, C-reactive protein; IL, Interleukin; LPS, lipopolysaccharide; MET, metabolic equivalent; MLM, multilevel models; WL, waitlist control group.

Values are mean (s.d.).
